# Abstract not submitted for publication

**DOI:** 10.1186/1532-429X-14-S1-T7

**Published:** 2012-02-01

## Abstract not submitted for publication

Abstract not submitted for publication

